# Key summary of German national guideline for adult patients with nosocomial pneumonia- Update 2024 Funding number at the Federal Joint Committee (G-BA): 01VSF22007

**DOI:** 10.1007/s15010-024-02358-y

**Published:** 2024-08-08

**Authors:** Jessica Rademacher, Santiago Ewig, Béatrice Grabein, Irit Nachtigall, Marianne Abele-Horn, Maria Deja, Martina Gaßner, Sören Gatermann, Christine Geffers, Herwig Gerlach, Stefan Hagel, Claus Peter Heußel, Stefan Kluge, Martin Kolditz, Evelyn Kramme, Hilmar Kühl, Marcus Panning, Peter-Michael Rath, Gernot Rohde, Bernhard Schaaf, Helmut J. F. Salzer, Dierk Schreiter, Hans Schweisfurth, Susanne Unverzagt, Markus A. Weigand, Tobias Welte, Mathias W. Pletz

**Affiliations:** 1 Department of Respiratory Medicine and Infectious Diseases, German Centre of Lung Research (DZL), Hannover Medical School, Hannover, Germany; 2 Department of Respiratory and Infectious Diseases, Thoraxzentrum Ruhrgebiet, EVK Herne and Augusta-Kranken-Anstalt Bochum, Bochum, Germany; 3 LMU Hospital, Clinical Microbiology and Hospital Hygiene, Munich, Germany; 4 Division of Infectious Diseases and Infection Prevention, Helios Hospital Emil-Von-Behring, Berlin, Germany; 5 Institute for Hygiene and Microbiology, University of Würzburg, Würzburg, Germany; 6 Department of Anesthesiology and Intensive Care Medicine, University Medical Center Schleswig-Holstein, Berlin, Lübeck, Germany; 7 Department of Anaesthesiology and Intensive Care Medicine, Charité, Universitätsmedizin Berlin, corporate member of Freie Universität Berlin and Humboldt-Universität Zu Berlin, Berlin, Germany; 8 National Reference Centre for Multidrug-Resistant Gram-Negative Bacteria, Department of Medical Microbiology, Ruhr-University Bochum, Bochum, Germany; 9 Charité, Universitätsmedizin Berlin, corporate member of Freie Universität Berlin and Humboldt-Universität Zu Berlin, Institute of Hygiene and Environmental Medicine, Berlin, Germany; 10 Department for Anaesthesia, Intensive Care Medicine and Pain Management, Vivantes-Klinikum Neukoelln, Berlin, Germany; 11 Institute for Infectious Diseases and Infection Control, Jena University Hospital-Friedrich Schiller University Jena, Jena, Germany; 12 Diagnostic and Interventional Radiology, Heidelberg University Hospital, Heidelberg, Germany; 13 Department of Intensive Care, University Hospital Hamburg-Eppendorf, Hamburg, Germany; 14 Division of Pulmonology, Medical Department 1, University Hospital of TU Dresden, Dresden, Germany; 15 Department of Infectious Diseases and Microbiology, University of Lübeck and University Hospital Schleswig-Holstein, Campus Lübeck, Germany; 16 Department of Radiology, St. Bernhard-Hospital Kamp-Lintfort, Bürgermeister-Schmelzing-Str. 90, 47475 Kamp-Lintfort, Germany; 17 Institute of Virology, Medical Center, University of Freiburg, Faculty of Medicine, University of Freiburg, Freiburg, Germany; 18 Institute for Medical Microbiology, University Medicine Essen, Essen, Germany; 19 Department of Respiratory Medicine, Goethe University Frankfurt, University Hospital, Frankfurt/Main, Germany; 20 Department of Respiratory Medicine and Infectious Diseases, Klinikum Dortmund, Dortmund, Germany; 21 Division of Infectious Diseases and Tropical Medicine, Department of Internal Medicine-Pneumology, Kepler University Hospital, Medical Faculty, Johannes Kepler University, Linz, Austria; 22 Department of Intensive Care Medicine, Helios Park Clinic, Leipzig, Germany; 23 Institute for Pulmonary Research (IPR), Cottbus, Germany; 24 Institute of General Practice and Family Medicine, Martin Luther University Halle-Wittenberg, Halle (Saale), Germany; 25 Department of Anesthesiology, Medical Faculty Heidelberg, Heidelberg University, Heidelberg, Germany

**Keywords:** Nosocomial pneumonia, Ventilator-associated pneumonia, German guideline, Antimicrobial stewardship, Septic shock

## Abstract

**Purpose:**

This executive summary of a German national guideline aims to provide the most relevant evidence-based recommendations on the diagnosis and treatment of nosocomial pneumonia.

**Methods:**

The guideline made use of a systematic assessment and decision process using evidence to decision framework (GRADE). Recommendations were consented by an interdisciplinary panel. Evidence analysis and interpretation was supported by the German innovation fund providing extensive literature searches and (meta-) analyses by an independent methodologist. For this executive summary, selected key recommendations are presented including the quality of evidence and rationale for the level of recommendation.

**Results:**

The original guideline contains 26 recommendations for the diagnosis and treatment of adults with nosocomial pneumonia, thirteen of which are based on systematic review and/or meta-analysis, while the other 13 represent consensus expert opinion. For this key summary, we present 11 most relevant for everyday clinical practice key recommendations with evidence overview and rationale, of which two are expert consensus and 9 evidence-based (4 strong, 5 weak and 2 open recommendations). For the management of nosocomial pneumonia patients should be divided in those with and without risk factors for multidrug-resistant pathogens and/or *Pseudomonas aeruginosa*. Bacterial multiplex-polymerase chain reaction (PCR) should not be used routinely. Bronchoscopic diagnosis is not considered superior to´non-bronchoscopic sampling in terms of main outcomes. Only patients with septic shock and the presence of an additional risk factor for multidrug-resistant pathogens (MDRP) should receive empiric combination therapy. In clinically stabilized patients, antibiotic therapy should be de-escalated and focused. In critically ill patients, prolonged application of suitable beta-lactam antibiotics should be preferred. Therapy duration is suggested for 7–8 days. Procalcitonin (PCT) based algorithm might be used to shorten the duration of antibiotic treatment. Patients on the intensive care unit (ICU) are at risk for invasive pulmonary aspergillosis (IPA). Diagnostics for *Aspergillus* should be performed with an antigen test from bronchial lavage fluid.

**Conclusion:**

The current guideline focuses on German epidemiology and standards of care. It should be a guide for the current treatment and management of nosocomial pneumonia in Germany.

**Supplementary Information:**

The online version contains supplementary material available at 10.1007/s15010-024-02358-y.

## Background

Nosocomial pneumonia is one of the most common nosocomial infections in Europe. According to data from the first European prevalence survey in 2011, pneumonia or lower respiratory tract infection accounts for 26% of all infections that develop during an inpatient stay [1]. In epidemiological studies, such as the European prevalence survey, pneumonia and lower respiratory tract infections are usually combined, as the information available in the survey does not always allow an exact distinction to be made. In intensive care units (ICU), pneumonia/lower respiratory tract infections even account for more than 40% of all nosocomial infections [2]. The new prevalence survey in 2016 confirmed pneumonia/lower respiratory tract infection as the most common nosocomial infection in Germany [3]. Thirty-three percent [4] and 35% [5] of nosocomial pneumonias are associated with mechanical ventilation in Europe and Germany, respectively.

A study on the relevance of nosocomial infections, which used European prevalence data and data on the consequences of nosocomial infections from the international literature, also identified nosocomial pneumonia as the most consequential type of infection [6]. For this purpose, disability-adjusted life years (DALYs) were calculated. According to this, nosocomial pneumonia alone causes 169 DALYs per 100,000 inhabitants in Europe and is therefore responsible for a third of the "disability-adjusted life-year losses" caused by nosocomial infections.

To provide a clinical treatment guideline that includes a profound evaluation of changing evidence, the scientific medical societies involved in the care of nosocomial pneumonia conducted an update of the guideline from 2017 [7]. Here, we present 11 key recommendations, that are most important for everyday clinical practice. The full-length guideline and evidence report are available in German language. https://register.awmf.org/de/leitlinien/detail/020-013

## Methods

### Aims of the guideline

The AMWF S3 guideline aims to provide a comprehensive overview of evidence-based recommendations on diagnostic and treatment of nosocomial pneumonia. The guideline addresses physicians involved in the inpatient care of adult patients with nosocomial pneumonia. This compendium highlights the key diagnostic and treatment recommendations of the guideline that are most important for clinical care.

### Determination of guideline questions

The guideline group comprised 26 delegates from 14 participating scientific medical societies and organizations, as well as a patient representative (appendix 1). The methodology used to create this guideline based on the current version of the AWMF guidelines https://www.awmf.org/regelwerk/. For all evidence-based recommendations, systematic searches for evidence-based guidelines, systematic reviews and primary studies were carried out based on 11 clinically relevant questions (appendix 2).

### Critical evaluation of the evidence

All references from the systematic search until August 2023 and additional studies (e.g. major RCTs that appeared after pre-defined search period deadline, see below) provided by the guideline group were screened on the basis of title, abstract and keywords by the methodologist (SU). The selection criteria for the target population, study design, comparisons and endpoints were agreed with the guideline group. Only studies in full text in English or German with the highest available level of evidence (systematic reviews, evidence-based guidelines, RCTs or cohort studies with confounder adjustment) were included. The methodological quality of the included studies was assessed using validated instruments depending on respective study designs [8, 9].

Evidence tables were created in accordance with the AWMF guidelines for summarizing study characteristics and results, and main information on all identified systematic reviews and meta-analyses as well as evidence-based guidelines were extracted. In addition, the authors’ conclusions were extracted, whereby it was checked whether the conclusion could be derived from the results. This was followed by an overall assessment by the reviewer, from which the evidence level of the individual systematic reviews was derived on the basis of the Oxford criteria [10]. The level of evidence was based on the study design and was downgraded by half a category (e.g. from 1 to 1-) in the case of moderate limitations in study quality, low precision of effect estimates, inconsistencies and indirectness and by one category (e.g. from 1 to 2) in the case of serious limitations or several limitations. Systematic reviews based on non-randomized studies were downgraded by one level of evidence (from 1 to 2). For each extracted study, a summarized assessment was performed, which included the conclusions of the study and the reviewer on the methodological quality of the studies.

The assessment of the quality of the evidence is based on the Cochrane Handbook [11] modified according to GRADE [12]. For all research questions, the evidence for all critical outcomes from all identified studies was summarized in an evidence profile and the quality of the evidence was assessed. This first describes the confidence in the results for each endpoint and across studies and then summarizes the results for the research question and is based on the design of the included studies, study limitations, the risk of publication bias, the precision and consistency of the effects and the transferability to the specified research question. Confidence in the results decreases from high to very low and reflect the confidence that the effect estimates are adequate to support a particular recommendation.

### Preparation of recommendations

All members of the panel had access to the evidence profiles in preparation for the consensus conferences. During three consensus conferences, evidence profiles were presented by a methodologist (SU) responsible for the respective evidence profile before results were discussed in plenary under neutral moderation. Based on the evidence profiles a structured evaluation of the respective diagnostic and treatment strategy was performed that included an appraisal of the following criteria by the guideline group: balance of desirable and undesirable effects, patient preferences, resources, equity, acceptability, and feasibility. Based on the evidence profile and overall assessment, recommendations were written and graded according to the AWMF standards (Strong recommendation- we recommend, conditional recommendation- we suggest, and recommendation open)https://www.awmf.org/regelwerk/.

For each recommendation delegates per medical society and organization had to vote (agree, disagree, abstention). Guideline group members with conflicts of interest were excluded from the respective voting. A strong consensus (agreement > 95%) was achieved for 10 out of 11 key recommendations presented in this executive summary while a consensus (agreement > 75%, but < 95%) was achieved for 1 of them. For each recommendation, background information was summarized by working groups within the guideline group to describe available evidence and rationale for the chosen grading to make the decision process as transparent as possible. Contents were finally reviewed by all members of the guideline group and officially validated by the participating medical societies and organizations.

### Expert consensus

Statements/recommendations for which the guideline group decided to work on the basis of expert consensus were labelled as expert consensus. No systematic literature search or assessment of the quality of the evidence was carried out for these recommendations. The studies cited in the background texts were selected by the participating experts. In the case of recommendations based on expert consensus, no quality levels or letters to describe the quality of the evidence and the level of recommendation were specified.

### Key recommendations

#### Pathogen spectrum and resistance

Risk factors for multidrug-resistant pathogens and/or *Pseudomonas aeruginosa*

For the initial, empiric antimicrobial therapy of nosocomial pneumonia, we recommend a distinction between patients with and without risk factors for multidrug-resistant pathogens and/or *Pseudomonas aeruginosa* (Table 1).

**Table 1:** Therapy-relevant risk factors for multi-resistant infectious pathogens in nosocomial pneumonia

Antimicrobial therapy (> 24 h) in the last 30 days
Hospitalization ≥ 5 days before onset of pneumonia
Colonization by gram-negative MDRP or MRSA
Septic shock
ARDS
Hemodialysis
Medical care in a high-prevalence country for Gram-negative MDRP and MRSA within the last 12 months
Additional risk factors for *P. aeruginosa*
Structural lung disease (advanced COPD, bronchiectasis)
Known colonization by *P. aeruginosa*

The pathogen spectrum and the resistance situation of the respective ward/facility should be recorded and presented at intervals of 6–12 months and decisions on empiric antibiotic therapy should be based on these data.

Strong recommendation, expert opinion, strong consensus

Evidence overview and rationale: the frequency of infections with multidrug-resistant pathogens (MDRP) and/or *Pseudomonas aeruginosa* (PA) depends on the presence of risk factors (Table 1). Due to the diverse intrinsic resistances of *Pseudomonas aeruginosa*, particularly against many beta-lactams, and the alignment of its risk factors for selection with those of pathogens with acquired multi-resistances, it is classified into the group "patients at risk for multi-resistant pathogens”. For pragmatic reasons, no distinction is made between wild-type *Pseudomonas aeruginosa* and *Pseudomonas aeruginosa* with additional acquired resistances.

A large number of studies [13–15] have examined the significance of individual risk factors for the detection of MDRP, primarily in ventilator-associated pneumonia (VAP). Previous antibiotic treatment (OR 13.5) and mechanical ventilation duration of > 7 days (OR 6.0) [13] were the strongest risk factor for MDRP according to a multivariate analysis [13]. Another study identified the highest MDRP risk for patients who had been treated with two different classes of antibiotics prior to pneumonia [14]. Others found that "potentially resistant bacteria" (*P. aeruginosa*, *Acinetobacter spp*., *S. maltophilia*, MRSA) were detected in 61.5% of cases and resistant bacteria in 30.3% of cases treated with intravenous antibiotics in the last month since onset of pneumonia compared to 17.8% and 6.7% in patients without prior antibiotic therapy [15]. In some studies, the severity of the disease (septic shock, acute organ dysfunction, ARDS) was only univariately associated with the detection of MDRP in VAP [13, 16]. The European guideline emphasizes serious illnesses such as septic shock, ARDS and a high local rate of MDRP (> 25%) as well as individual factors as risk factors for MDRP [17]. Pre-existing colonization with MDRP or a high local rate, regionally or in hospitals, is a further risk factor for pneumonia with MDRP. Accordingly, 5–25% of patients in intensive care units were carriers of ESBL-positive enterobacterales; 5–20% of patients colonized with ESBL developed pneumonia due to ESBL-forming bacteria (VAP) [18]. The presence of severe structural lung disease (severe COPD, bronchiectasis) was identified as a specific risk factor for nosocomial pneumonia caused by *P. aeruginosa* in addition to a proven chronic respiratory tract infection [19–22]. In another study, intensive care hospitalization of more than 29 days was also a major risk factor [23]. When MRSA colonization was detected, the positive predictive value for MRSA pneumonia in studies was between 18 and 35% [24–26].

The weighting of these factors cannot be precisely quantified. The risk depends on the susceptibility of the patient, the duration and intensity of exposure to individual risk factors, the interaction of several factors and the local pathogen epidemiology (probability of acquiring MDRP from the hospital environment). For this reason, local susceptibility data should be used for treatment planning. Ideally, the survey should be based on the pathogens detected in HAP, but at least on those detected in respiratory materials.

### Diagnostic approach

#### Microbiology

The regular use of bacterial multiplex PCR systems in patients with suspected nosocomial pneumonia cannot be recommended

*Quality of evidence for impact on mortality, ventilation duration, antibiotic days, time until de-escalation: very low; recommendation open, strong consensus*

**Evidence overview and rationale**: to date, there are only a few studies that have prospectively investigated the impact of molecular biological diagnostics with regard to antibiotic consumption, ventilation/recovery time and mortality. Three RCTs with overall 1004 patients were included in the assessment [27–29]. In a monocentric, prospective study, 605 unselected non-intubated patients with radiologically diagnosed pneumonia were examined to determine whether the results of the Curetis unyvero P50 assay from bronchoalveolar lavage fluid (BALF) have an influence on the length of hospital stay and the use of antibiotics. Fifty four percent of the patients were immunocompromised, most of them with a post lung transplant condition. Although the detection frequency of the molecular biological method was significantly higher than that of the cultural analysis (82% vs. 56%, especially *H. influenzae*, *A. baumannii*), the molecular biological results had no influence on the length of hospital stay and the administration (duration and number) of antibiotics [29]. Another multicenter, randomized controlled trial including 23% patients with nosocomial pneumonia showed a relevant reduction in the duration of antibiotic treatment by 34 h [27], which could not be confirmed in another study with critically ill ventilated SARS-Cov2 pneumonia patients [28]. Differences in mortality could not be shown in any of these studies. Information on de-escalation was only available from the PCR group of one study [27]. Based on available evidence to date, the guideline group recommends against routinely use of bacterial multiplex PCR systems. Potential harms of the use of multiplex PCR assays include cost and the potential for inappropriate escalation of antibiotics based on a false-positive PCR result. The results of PCR for resistance genes were not reliable.

Mycological diagnostics

We suggest rapid and targeted diagnostics for *Aspergillus* in patients with nosocomial pneumonia on the ICU with risk factors for invasive pulmonary aspergillosis (IPA) (e.g. steroid therapy, COPD, liver cirrhosis, malnutrition, burns, diabetes, severe influenza or COVID-19 infection), even in the absence of severe immunosuppression such as neutropenia, if IPA is suspected.

For *Aspergillus* detection, at least one antigen test for galactomannan (GM) (limit value ODI >  = 1.0) from bronchoalveolar lavage (BAL) and, if necessary, additional microbiological procedures should be performed.

Quality of evidence for mortality: very low; conditional recommendation, strong consensus

Evidence overview and rationale: two systematic reviews on diagnostic quality as well as two systematic reviews and two randomized studies on treatment strategies were identified [30–35]. Laboratory tests consistently indicated a better diagnostic performance of BALF GM than serum GM, and a suboptimal specificity of BALF 1,3 beta-D-glucan (BDG) and serum BDG. Only indirect evidence was available from critically ill organ transplant patients or neutropenic adults with haematological malignancies [30, 35]. The efficacy and safety of prompt adequate treatment with antifungal agents in adult patients with nosocomial pneumonia and invasive aspergillosis was found in two systematic reviews [32, 34] and two additional new randomized controlled trials [31, 33]. Effective treatment strategies can reduce mortality but existing data are limited to target populations only. Due to indirectness, insufficient precision and other limitations, the evidence was summarized as very low. The authors’ aim in making this recommendation was to raise the awareness for invasive Aspergillosis in patients with nosocomial pneumonia, given that the according risk factors are not identical to ‘classical’ risk factors such as neutropenia.

Diagnosis of nosocomial pneumonia

A bronchoscopic microbiological sampling is not superior to a non-bronchoscopic microbiological sampling in VAP in terms of important outcomes. Therefore, the decision about a bronchoscopic sampling should be made depending on local logistics, differential diagnostic considerations, and possible therapeutic aspects of an endoscopic examination.

Quality of evidence for mortality, adequate anti-infective therapy: high; for antibiotic days, ventilator days: moderate; strong recommendation, strong consensus

Evidence overview and rationale: an evaluation of the evidence comparing the efficacy and safety of bronchoscopic sampling versus tracheobronchial aspirates for the diagnosis of nosocomial pneumonia (VAP) in terms of important outcomes such as the rate of adequate treatment, duration of antibiotic therapy, duration of ventilation and mortality was performed. A systematic review and five randomized primary studies comparing results after invasive and non-invasive diagnostics were identified [36–41]. All studies were published between 1998 and 2006 and included in the systematic review of Benton et al. published in 2014. There was no evidence that the use of invasive strategies compared to non-invasive results in reduced mortality, reduced time in ICU and on mechanical ventilation, or higher rates of antibiotic change when compared to quantitative cultures of tracheobronchial aspirates in patients with VAP. Nevertheless, the importance of quantitative culture of BALF has recently been affirmed [42]. The lack of neutrophilia in the BALF has a high negative predictive value. The number of intracellular pathogens also provides important information [42]. The visualization of distal purulent secretions and the persistence of distal secretions during expiration have been described as independent predictors of pneumonia [43]. Finally, BALF enables additional testing for viruses (most recently SARS-CoV-2, but also influenza and RSV) and fungi (especially Aspergillus spp., here also by determining the galactomannan), which have recently become more important in the context of nosocomial pneumonia.

Overall, non-invasively obtained and usually readily available tracheobronchial aspirates remain an equally valid material for the diagnosis of VAP, while in selected patients bronchoscopic sampling with (semi)quantitative cultures may provide an advantage.

### Therapy

#### Antibacterial therapy

We suggest the use of aminopenicillins with beta-lactamase inhibitors or group 3a cephalosporins (i.e. ceftriaxone or cefotaxime) in patients without an increased risk of MDRP/PA (Table 1). Fluoroquinolones with pneumococcal activity (i.e. moxifloxacin or levofloxacin) can be used as a secondary option.

Piperacillin/tazobactam, cefepime or meropenem should be used in patients with an increased risk of MDRP/PA (Table 1). Potential combination substances (see 3.2) include antipseudomonal fluoroquinolones, fosfomycin or aminoglycosides.

The choice of substance -or combination in selected patients- should be made based on local pathogen spectrum and resistance profiles.

Strong recommendation, expert opinion, strong consensus

**Fig. 1:**
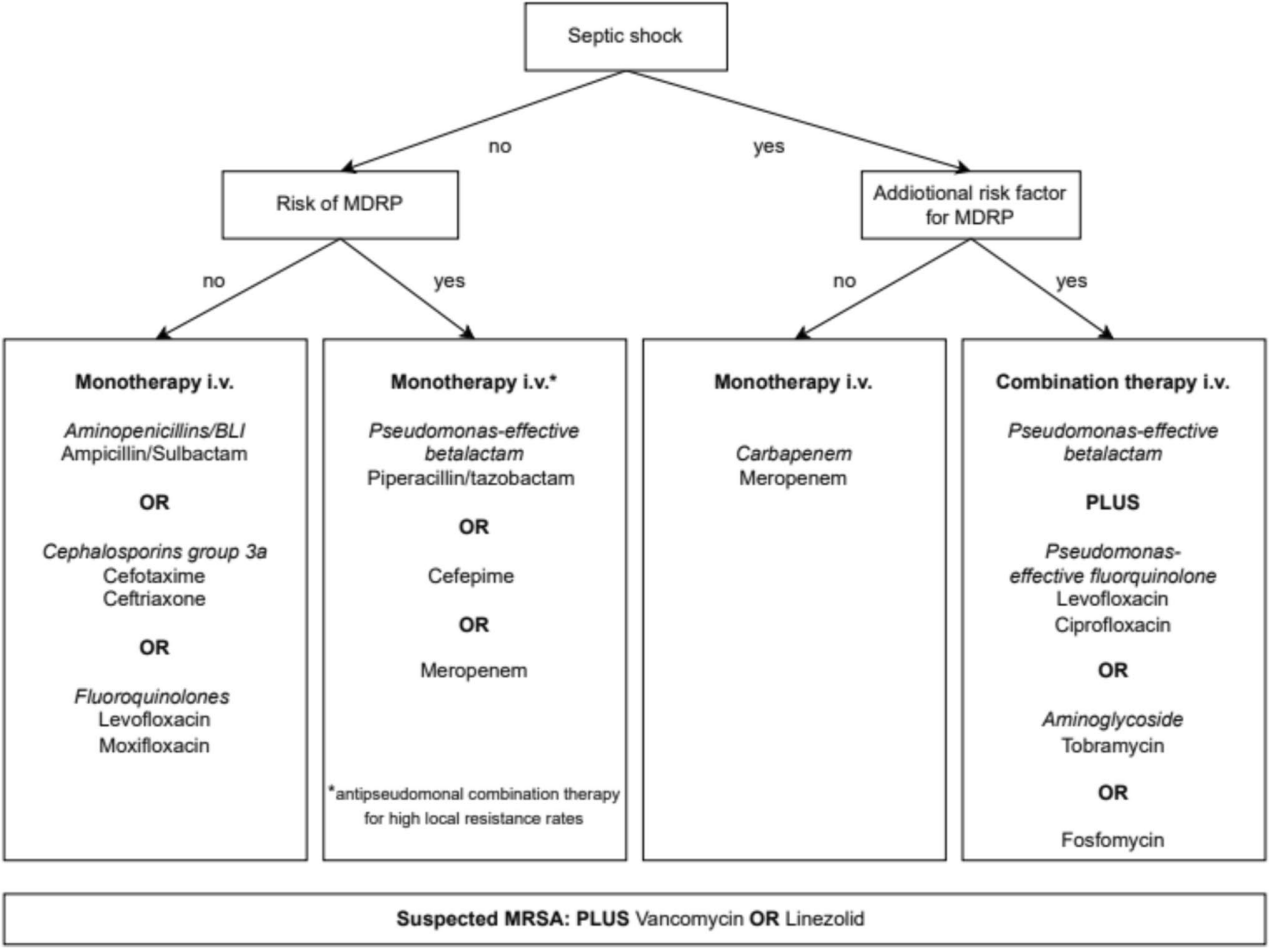
Flow chart for empiric therapy of nosocomial pneumonia. *MDRP* multidrug-resistant pathogens

Evidence overview and rationale: Data on pathogen spectrum and treatment of nosocomial pneumonia in patients without invasive ventilation and MDRP/PA risk factors is very limited. The patient population is more heterogeneous and the pathogen detection rate is significantly lower than for VAP. Piperacillin/tazobactam, cephalosporins of groups 3a (i.e. ceftriaxone, cefotaxime) and 3b (i.e. ceftazidime), carbapenems and moxifloxacin have been tested and were not found to be superior in terms of mortality or clinical treatment success [44–46]. Thus, in patients with low risk of MDRP/PA (Table 1), treatment with a limited spectrum antibiotic is recommended (Fig. 1). The local pathogen spectrum and resistance data should be taken into account when selecting the substance. The combination with macrolides as anti-inflammatory strategy has not been sufficiently investigated in HAP or VAP. Regular coverage of Legionella pneumophila, Mycoplasma pneumoniae and Chlamydophilia spp. is not required [47].

The term Gram-negative MDRP has no standardized definition and refers to bacterial strains with resistance to several classes of antibiotics, among others, the following bacterial species: *E. coli*, *Klebsiella pneumoniae*, *P. aeruginosa* and *Acinetobacter baumanii*. The clinical evidence for the use of specific antibiotics VAP is low. Piperacillin/tazobactam, pseudomonas-effective cephalosporins, pseudomonas-effective carbapenems and the fluoroquinolones ciprofloxacin and levofloxacin were tested in monotherapy or combination therapy in several studies without finding any superiority of a substance or combination in terms of mortality [48]. Most of the data were collected as part of approval studies with the aim of equivalence in well-selected cohorts. Due to these limitations, no advantage of a substance or a regimen over another could be shown with regard to mortality. With regard to treatment failure, also no significant differences were found in most studies and in a meta-analysis that included over 7000 patients with VAP [49]. However, ceftazidime monotherapy performed worse than meropenem or piperacillin/tazobactam in terms of clinical response in several studies [49, 50]. The substance has insufficient activity against *S. aureus* and pneumococci, the most frequent Gram-positive pathogens in nosocomial pneumonia and should therefore not be administered as empiric therapy.

If MRSA infection is suspected in the presence of sepsis or septic shock, vancomycin or linezolid should be added. If colonization with an ESBL producing and/or another multidrug-resistant Gram-negative strain (MRGN) is known, a treatment regimen should be selected that also includes the corresponding ESBL and/or MRGN. The recommendations given in Fig. 1 take into account the current epidemiological data in Germany.

#### Mono-versus combination therapy

We suggest an initial empiric combination therapy in patients with septic shock AND the presence of at least one additional risk factor for MDRP (Table 1 / Fig. 1).

In patients with septic shock and an increased risk of *P. aeruginosa* (Table 1), *P. aeruginosa*-effective combination therapy should be given until the results of the susceptibility test are available.

Quality of evidence for mortality: very low; conditional recommendation, strong consensus

Evidence overview and rationale: An evidence-based guideline, 8 systematic reviews/meta-analyses, and an RCT [51–58] evaluated combination therapies for nosocomial pneumonia. These studies covered various patient groups and substances (pneumonia, MDRP infections, *P. aeruginosa* bacteremia, septic shock presence, Carbapenem resistant Gram-negative pathogens, tigecycline or colistin for MDRP). One meta-analysis found no benefit in mortality or other outcomes for severe sepsis combination therapy [58]. Another analysis of 50 studies indicated a survival benefit for septic shock patients, but these studies had several methodological limitations [54]. In non-severe cases, combination therapy correlated with higher mortality, possibly due to direct toxicity, resistance, or *Clostridioides difficile* infections [59]. A study suggested an advantage of initial combination therapy for VAP with *P. aeruginosa* when it prevented ineffective monotherapy [60]. However, older studies found no benefit for combination therapy for *P. aeruginosa* bloodstream infections and pneumonia [53]. An observational study reported no survival benefit from adding aminoglycoside to beta-lactam antibiotic therapy for *P. aeruginosa* infections [61]. The CLSI argues against use of aminoglycosides for *P. aeruginosa.* No safe aminoglycoside-dosing regimen was predicted to achieve bacterial 1- or 2-log killing, regardless of the breakpoint applied. Comparing meropenem monotherapy to meropenem plus ciprofloxacin showed no mortality difference, except in a subgroup with better microbiological response for MDRP infections [62]. Low-quality evidence indicates no general advantage for combination therapy, but specific therapies and groups may show lower mortality. Overall, disease severity, local resistance rates, patient risk profiles, and potential toxicity should guide the administration of combination therapy and substance choice.

#### Prolonged infusion of beta-lactams

We suggest prolonged application of suitable beta-lactam antibiotics after the initial loading dose in critically ill patients.

Quality of evidence for mortality, clinical cure: low; conditional recommendation, strong consensus

Evidence overview and rationale: five recent systematic reviews were identified that examined the efficacy and safety of prolonged administration (3–4 h or continuous infusion) with intermittent bolus administration of beta-lactam antibiotics on mortality, clinical recovery, side effects and the occurrence of antibiotic-resistant bacteria [63–67]. All papers reported mortality and clinical cure, especially in critically ill ICU patients with different underlying infections, but in the majority with a pulmonary focus. Only one systematic review reported adverse events and antibiotic-resistance [65]. Low-quality evidence shows reduced mortality and improved clinical recovery with prolonged use of beta-lactam antibiotics in critically ill patients. Therefore, it is recommended that prolonged infusion of beta-lactam should be used preferred in patients with sepsis and septic shock. Due to the small number of studies, no statement can be made regarding the occurrence of side effects and antibiotic-resistant bacteria.

#### Inhaled antimicrobial therapy

We do not suggest routinely inhaled antibiotic therapy in addition to systemic therapy.

Quality of evidence for mortality, antibiotic days: moderate; conditional recommendation, strong consensus

In the presence of multi-resistant Gram-negative pathogens that are only sensitive to colistin and/or aminoglycosides, supplementary inhalation therapy with suitable nebulizers should be considered in addition to systemic antibiotic therapy.

Quality of evidence for clinical response: very low; conditional recommendation, strong consensus

Evidence overview and rationale: four reviews [68–71] evaluating the evidence on the effectiveness of inhaled antibiotic therapy in patients with VAP on mortality, eradication rate, length of stay and duration of ventilation were identified. Moderate-quality evidence showed no effect on mortality and duration of treatment, but improved eradication rates with inhaled antibiotic therapy. An increased incidence of renal side effects was not seen. In one metaanalysis, no survival benefit (relative risk (RR) 1.00, 95% confidence interval (CI) 0.82–1.21) but a higher clinical cure rate (RR 1.13, 95% CI 1.02–1.26) and more frequent microbiological eradication (RR 1.45, 95% CI 1.19–1.76) were observed in therapy combined with inhaled antibiotics compared to intravenous therapy alone [69].

The high local concentrations in the bronchial system after inhalation of antibiotics could be particularly beneficial for infections with MDRP. Local application reduces the selection pressure on the intestinal microbiome and may be beneficial in the case of pre-existing renal insufficiency. The penetration of aerosolized antibiotics into the affected lung parenchyma is unsure, especially in ventilated patients with severe lung infections, so that the deposition of the inhaled drug may not be sufficient [72]. Furthermore, inhaled antibiotics were associated with an increased risk of bronchospasm.

An improved outcome (clinical response, microbiological eradication and infection-associated mortality) was found with additional inhaled colistin application, albeit with a low level of evidence and no effect on overall mortality [70]. A further meta-analysis based on 12 studies with 812 patients found an advantage in terms of clinical response, however, the analysis was underpowered [73].

The current IDSA/ATS guideline [74] recommends inhaled antibiotic therapy in addition to systemic antibiotic therapy for HAP/VAP caused by carbapenem-resistant Gram-negative pathogens that are only sensitive to aminoglycosides and polymyxins, or if *A. baumannii* with sensitivity exclusively to polymyxins is detected.

Inhaled therapy may be particularly useful for patients who cannot be treated adequately systemically or only at the risk of considerable toxicity. In a prospective observational study, patients with VAP and evidence of sensitive *P. aeruginosa* or *A. baumannii* and intravenous therapy were compared with patients and evidence of multidrug-resistant *P. aeruginosa* or *A. baumannii* with high-dose inhaled colistin therapy (3 × 5 million IU) with and without intravenous aminoglycoside over 3 days in terms of clinical recovery and mortality [75]. The group of multidrug-resistant pathogens was not inferior to the systemic therapy when colistin was inhaled.

#### De-escalation and focusing

We recommend de-escalation in patients with clinical treatment response even without pathogen detection.

In patients with microbiological evidence of a relevant pathogen, therapy should be focused.

Quality of evidence for mortality: moderate; eradication rate, ventilator days, length of stay: very low; strong recommendation, strong consensus

Evidence overview and rationale: Five systematic reviews and eight cohort studies were identified to evaluate the efficacy and safety of de-escalation and focusing therapy [76–83]. The definition of de-escalation was applied inconsistently in the studies. De-escalation from combination to monotherapy and from broad-spectrum to targeted therapy according to microbiological results was best investigated. The different de-escalation options are not separated in all studies. In a multicenter prospective observational study, 244 critically ill patients with nosocomial pneumonia in 24 intensive care units were included [77]. Focused therapy could have been carried out in 94 patients on the basis of the pathogen detected; in fact, it was carried out in 56 patients. A comparison of the groups showed a reduced mortality after focused therapy. In a second study from Canada and the USA, focused therapy resulted in a better outcome with a lower application density of broad-spectrum antibiotics [82]. In VAP due to *P. aeruginosa*, de-escalation to monotherapy after receipt of the antibiogram did not lead to increased mortality in a monocentric retrospective study [79]. Similar results were shown in a prospective cohort analysis of patients with bacteremia with *P. aeruginosa* [84].

On the other hand, de-escalation to an effective monotherapy was not a disadvantage compared to continued administration of a combination. It was also shown in surgical intensive care units that de-escalation did not lead to increased mortality in critically ill patients [78]. The main focus was on de-escalation from combination to monotherapy. In another smaller retrospective study of patients with VAP, de-escalation from broad-spectrum to narrower therapy did not result in increased mortality [80]. A similar study, also with a retrospective design, showed a reduction in the duration of treatment [81]. Thus, there was no difference in mortality both for de-escalation based on clinical response and on the basis of available microbiological findings. There was also no difference in ventilation duration, length of stay, antibiotic days and the occurrence of recurrent infections.

There are no sufficient studies which adressed whether a selection of multi-resistant pathogens can be prevented by de-escalation.

#### Therapy duration

We suggest a therapy duration of 7–8 days if the patient responds well. In individual cases, longer treatment durations may be necessary (e.g. *S. aureus* bacteremia, non-remediable empyema, abscess).

Quality of evidence for mortality, length of stay, clinical cure: high; selection MDRP: moderate; recommendation, strong consensus

A PCT-based algorithm can be used in patients with nosocomial pneumonia to shorten the duration of antibiotic treatment.

Quality of evidence: mortality, antibiotic days: moderate; recommendation open, consensus

Evidence overview and rationale: Over the past two decades, several prospective, randomized, controlled trials have been conducted in patients with ventilator-associated pneumonia to compare shorter (7–8 days) versus longer (10–15 days) duration of therapy [85–89]. In a recent meta-analysis [90], which included these five studies with a total of 1069 patients with ventilator-associated pneumonia, a shorter duration of therapy did not differ from a longer duration of therapy with regard to the endpoints mortality, length of stay, relapse rate and the occurrence of multidrug-resistant pathogens in the treatment of ventilator-associated pneumonia. This also applies to the group of patients with HAP due to gram-negative non-fermenters. In three of the five included studies, a total of 340 patients with HAP due to Gram-negative non-fermenters (most of them with evidence of Pseudomonas aeruginosa) were examined using subgroup analyses. No significant difference was found with regard to the recurrence or relapse rate between the longer and shorter treatment duration, nor was there a significant difference when considering 28-day mortality.

There are no data on the duration of treatment of nosocomial pneumonia in non-ventilated patients.

It should be noted that the number of patients included who had severe ARDS or septic shock was low. Patients with structural lung disease, such as bronchiectasis, as well as those with lung abscess or empyema were regularly excluded [91]. In the iDIAPASON study, patients with documented cultural evidence of Pseudomonas aeruginosa in respiratory materials before the start of HAP were excluded [85].

Nevertheless, treatment duration of seven to eight days appears to be sufficient in patients with nosocomial pneumonia and treatment response. In patients with evidence of *P. aeruginosa* as the causative agent of HAP, treatment duration of seven to eight days can also be considered.

In patients with structural lung disease (bronchiectasis) or lung abscesses, as well as patients with severe ARDS and/or septic shock, the duration of therapy should be determined individually. Another exception is HAP caused by bacteriemic *S.aureus*. This is classified as complicated *S. aureus* bacteremia and is usually treated for at least four weeks [92, 93].

*Procalcitonin- algorithm:* several prospective, randomized studies and two recent meta-analyses [94–99] have adressed this question. Primary studies confirm that PCT-based algorithms reduce antibiotic therapy duration within a defined PCT determination and response protocol, even including overruling by clinicans. Except for one study, who reported median therapy durations of 5 days (3–9) for the PCT-guided group versus 7 days (4–11) for the control [97], no study achieved less than seven days in the intervention group. In De Jong et al.’s study of 1575 sepsis and septic shock patients, mostly with pulmonary infections, also found lower mortality in the intervention group (20% vs. 27%, 6.6% difference, 95% CI 1.3–11.9) [97]. This difference persisted after one year (36% vs. 43%, 7.4% difference, 95% CI 1.3–13.8), potentially due to earlier diagnosis and treatment of non-bacterial infections and fewer adverse drug reactions from shorter treatments. However, the study’s limitation is that about half the patients had community-acquired infections, so results may not apply to nosocomial pneumonia.

Overall, PCT-based algorithms can reduce antibiotic therapy duration, but this moderate evidence is mainly based on older studies with treatment durations beyond 7–8 days. Consequently, the guideline group downgraded the recommendation from "suggest" to “may be considered to use." For practitioners with already short therapy durations, PCT algorithms may not further reduce treatment time. However, in clinics with traditionally longer treatments (> 8 days), a predefined PCT algorithm may help shorten therapy.

## Conclusions

Several clinical practice guidelines have been published for diagnosis and treatment of patients with nosocomial pneumonia. The most recent international guidelines are more than 6 years old (74,100).

These recommendations are based on a multidisciplinary approach, involving specialists from different healthcare systems and medical domains, and follow the GRADE approach.

The current recommendations (summarized in Table 2) is thought to benefit physicians dealing with the care of patients and to help standardize the current treatment and management of nosocomial pneumonia.

**Table 2:** Summary of recommendations with quality of evidence level

**∑** !!	1. For the initial, empiric antimicrobial therapy of nosocomial pneumonia, we recommend a distinction between patients with and without risk factors for multidrug-resistant pathogens and/or Pseudomonas aeruginosa (Table 1) The pathogen spectrum and the resistance situation of the respective ward/facility should be recorded and presented at intervals of 6–12 months and decisions on empiric antibiotic therapy should be based on these data
⊕ ⊝ ⊝ ⊝ o	2. The regular use of bacterial multiplex PCR systems in patients with suspected nosocomial pneumonia cannot be recommended
⊕ ⊝ ⊝ ⊝ !	3. We suggest rapid and targeted diagnostics for Aspergillus in patients with nosocomial pneumonia on the ICU with risk factors for invasive pulmonary aspergillosis (IPA) (e.g. steroid therapy, COPD, liver cirrhosis, malnutrition, burns, diabetes, severe influenza or COVID-19 infection), even in the absence of severe immunosuppression such as neutropenia, if IPA is suspected For Aspergillus detection, at least one antigen test for galactomannan (GM) (limit value ODI > = 1.0) from bronchoalveolar lavage (BAL) and, if necessary, additional microbiological procedures should be performed
⊕ ⊕ ⊕ ⊕ !!	4. A bronchoscopic microbiological sampling is not superior to a non-bronchoscopic microbiological sampling in VAP in terms of important outcomes. Therefore, the decision about a bronchoscopic sampling should be made depending on local logistics, differential diagnostic considerations, and possible therapeutic aspects of an endoscopic examination
**∑** !!	5. We suggest the use of aminopenicillins with beta-lactamase inhibitors or group 3a cephalosporins (i.e. ceftriaxone or cefotaxime) in patients without an increased risk of MDRP/PA (Table 1). Fluoroquinolones with pneumococcal activity (i.e. moxifloxacin or levofloxacin) can be used as a secondary option Piperacillin/tazobactam, cefepime or meropenem should be used in patients with an increased risk of MDRP/PA (Table 1). Potential combination substances (see 3.2) include antipseudomonal fluoroquinolones, fosfomycin or aminoglycosides The choice of substance -or combination in selected patients- should be made based on local pathogen spectrum and resistance profiles
⊕ ⊝ ⊝ ⊝ !	6. We suggest an initial empiric combination therapy in patients with septic shock AND the presence of at least one additional risk factor for MDRP (Table 1 / Fig. 1) In patients with septic shock and an increased risk of P. aeruginosa (Table 1), P. aeruginosa-effective combination therapy should be given until the results of the susceptibility test are available
⊕ ⊕ ⊝ ⊝ !	7. We suggest prolonged application of suitable beta-lactam antibiotics after the initial loading dose in critically ill patients
⊕ ⊝ ⊝ ⊝ !	8. We do not suggest routinely inhaled antibiotic therapy in addition to systemic therapy In the presence of multi-resistant Gram-negative pathogens that are only sensitive to colistin and/or aminoglycosides, supplementary inhalation therapy with suitable nebulizers should be considered in addition to systemic antibiotic therapy
⊕ ⊕ ⊕ ⊝ !!	9. We recommend de-escalation in patients with clinical treatment response even without pathogen detection In patients with microbiological evidence of a relevant pathogen, therapy should be focused
⊕ ⊕ ⊕ ⊕ !	10. We suggest a therapy duration of 7–8 days if the patient responds well. In individual cases, longer treatment durations may be necessary (e.g. S. aureus bacteremia, non-remediable empyema, abscess)
⊕ ⊕ ⊕ ⊝ o	11. A PCT-based algorithm can be used in patients with nosocomial pneumonia to shorten the duration of antibiotic treatment

⊕  ⊕  ⊕  ⊕ High quality of evidence

!!strong recommendation

⊕  ⊕  ⊕  ⊝ Moderate quality of evidence

! conditional recommendation

⊕  ⊕  ⊝  ⊝ Low quality of evidence

o recommendation open

⊕  ⊝  ⊝  ⊝ Very low quality of evidence

∑: Expert consensus

Implementation is obviously challenging, depending on the healthcare systems and resources allocated; however, these guidelines provide clear, focused, and concise recommendations.

## Supplementary Information

Below is the link to the electronic supplementary material.

Supplementary file1 (DOCX 14 KB)

Supplementary file2 (DOCX 15 KB)

## Data Availability

No datasets were generated or analysed during the current study.
