# Supplementary material for: Key summary of German national guideline for adult patients with nosocomial pneumonia- Update 2024 Funding number at the Federal Joint Committee (G-BA): 01VSF22007
Source: Infection. 2024 Aug 8;52(6):2531–45. doi: 10.1007/s15010-024-02358-y (PMC11621171; doi:10.1007/s15010-024-02358-y)
Supplement: Supplementary file 1 — Supplementary file1 (DOCX 14 KB) [file 15010_2024_2358_MOESM1_ESM.docx]

Appendix 1: Guideline group: delegates and represented German medical societies

| Medical society | Delegate |
| --- | --- |
| German Society of Hygiene and Microbiology (DGHM) | Prof. Dr. Abele-Horn |
| German Society of Anaesthesiology and Intensive Care Medicine (DGAI) | Prof. Dr. Maria Deja |
| German Respiratory Society (DGP)* | Prof. Dr. Santiago Ewig |
| German Society of Anaesthesiology and Intensive Care Medicine (DGAI) | Dr. Martina Gaßner |
| Paul Ehrlich Society for Infection Therapy e.V. (PEG) | Prof. Dr. Sören Gatermann |
| German Sepsis Society (DSG) | Prof. Dr. Christine Geffers |
| Paul Ehrlich Society for Infection Therapy e.V. (PEG) | Dr. Béatrice Grabein |
| German Society of Anaesthesiology and Intensive Care Medicine (DGAI) | Prof. Dr. Herwig Gerlach |
| German Society for Internal Medicine (DGIM) | PD Dr. Stefan Hagel |
| German Radiological Society (DRG) | Prof. Dr. Claus Peter Heußel |
| German Society for Medical Intensive Care Medicine and Emergency Medicine (DGIIN) | Prof. Dr. Stefan Kluge |
| German Respiratory Society (DGP)* | Prof. Dr. Martin Kolditz |
| German Society of Infectious Diseases (DGI) | Dr Evelyn Kramme |
| German Radiological Society (DRG) | Dr. Hilmar Kühl |
| German Society of Anaesthesiology and Intensive Care Medicine (DGAI) | Prof. Dr. Irit Nachtigall |
| German Society for Virology (GfV) | Prof. Dr. Markus Panning |
| German Society of Infectious Diseases (DGI) | Prof. Dr. Mathias W. Pletz |
| German Respiratory Society (DGP)* | PD Dr. Jessica Rademacher |
| German Society of Hygiene and Microbiology (DGHM) | Prof. Dr. Peter-Michael Rath |
| German Respiratory Society (DGP)* | Prof. Dr. Gernot Rohde |
| German Respiratory Society (DGP)* | PD Dr. Bernhard Schaaf |
| Chronic pulmonary aspergillosis network (CPAnet) | Prof. Dr. Helmut Salzer |
| German Society of Surgery (DGCH) | Dr. Dierk Schreiter |
| German Society for Internal Medicine (DGIM) | Prof. Dr. Hans Schweisfurth |
| German Sepsis Society (DSG) | Prof. Dr. Markus Weigand |
| Paul Ehrlich Society for Infection Therapy e.V. (PEG) | Prof. Dr. Tobias Welte |
| Patient organization | Delegate |
| German sepsis aid (DSH) | Prof. Dr. Frank Brunkhorst |

Guideline contributors and delegates of the medical societies are presented in alphabetic order

* Guideline leading medical society
