# Supplementary material for: Key summary of German national guideline for adult patients with nosocomial pneumonia- Update 2024 Funding number at the Federal Joint Committee (G-BA): 01VSF22007
Source: Infection. 2024 Aug 8;52(6):2531–45. doi: 10.1007/s15010-024-02358-y (PMC11621171; doi:10.1007/s15010-024-02358-y)
Supplement: Supplementary file 2 — Supplementary file2 (DOCX 15 KB) [file 15010_2024_2358_MOESM2_ESM.docx]

**Appendix 2: Objectives and clinically relevant questions**

Comprehensive improvement in the quality of care for patients with nosocomial pneumonia

- Ensuring a high level of adequate therapy
- Establishment of decision-making aids for diagnostics and therapy
- Optimizing the rational use of antibiotics with adequate antibiotic selection, dosage and duration of therapy in the sense of antimicrobial stewardship
- Avoiding the selection of resistant pathogens
- Reduction of hospital stay through shorter treatment durations with cost savings
- Reduction in mortality
- Evaluation and recommendation on the use of new (expensive) antibiotics for multi-resistant germs
- Greater focus on viruses as the cause of pneumonia and fungi as superinfections

Clinically relevant questions:

- Use of bacterial multiplex PCR techniques to improve the diagnosis of nosocomial pneumonia
- Which patients are at particular risk of infection with Aspergillus?
- Is invasive bronchoscopic sampling superior to non-bronchoscopic sampling?
- What is the value of calculated combination therapy for nosocomial pneumonia?
- Is which patients are prolonged application of beta-lactam antibiotics preferred?
- When is inhaled antibiotic therapy indicated?
- Is de-escalation and focusing therapy possible in HAP/VAP?
- What is the optimal duration of therapy and which markers can be used to control it?
